# Vanadate reduction by gram-positive fermentative bacteria isolated from deep-sea sediments on the northern Central Indian Ridge

**DOI:** 10.1371/journal.pone.0317320

**Published:** 2025-01-22

**Authors:** Bokyung Kim, Dong Kyun Woo, Juhwan Jeong, Min Sub Sim

**Affiliations:** School of Earth and Environmental Sciences, Seoul National University, Seoul, South Korea; National Chung Cheng University, Taiwan & Australian Center for Sustainable Development Research and Innovation (ACSDRI), AUSTRALIA

## Abstract

The oxidation states of vanadium determine its mobility and toxicity, and dissimilatory vanadate reduction has been reported in several microorganisms, highlighting the potential significance of this pathway in the remediation of vanadium contamination and the biogeochemical cycle. However, to date, most known microorganisms capable of reducing vanadate are Gram-negative respiratory bacteria belonging to the phylum Proteobacteria. In this study, we isolated *Tepidibacter mesophilus* strain VROV1 from deep-sea sediments on the northern Central Indian Ridge and investigated its ability to reduce vanadium and the impact of vanadate on its cellular metabolism. A series of culture experiments revealed that the isolated strain efficiently reduces V(V) to V(IV) during fermentation, even at mM levels, and this reduction involves a direct biological process rather than indirect reduction via metabolic products. Vanadium affects microbial carbon and nitrogen metabolism. Notably, in the presence of vanadate, alanine production decreases, suggesting that metabolic flux is diverted from the transamination reaction to vanadate reduction. *T*. *mesophilus* VROV1 is the second Gram-positive bacterium identified to reduce vanadium, following *Lactococcus raffinolactis*, but these bacteria belong to different classes: *T*. *mesophilus* is classified as *Clostridia*, whereas *L*. *raffinolactis* is classified as *Bacilli*. The specific rate of vanadate removal by VROV1 was as high as 2.8 pmol/cell/day, which is comparable to that of metal-reducing bacteria and markedly exceeds that of *L*. *raffinolactis*. Our findings expand the distribution of vanadate-reducing organisms within the bacterial domain. Given the wide range of natural habitats of *T*. *mesophilus* and its close relatives, we speculate that fermentative vanadate reduction may have a greater impact on the global biogeochemical cycle of vanadium than previously thought.

## Introduction

Vanadium is ubiquitous in nature, ranking as the second most common transition metal in seawater and the 19th most abundant element in the crust [1, 2]. Under Earth’s surface conditions, the valence state of vanadium (+3, +4, and +5) strongly influences the solubility of its species, thereby determining their mobility within various environments [3]. Recently, in addition to natural processes such as weathering and volcanism, anthropogenic activities have emerged as a significant source of vanadium inputs into aquatic environments because of the growing industrial demand for this element [4]. Vanadium is both beneficial and toxic to living organisms. It constitutes the active site of several enzymes, including nitrate reductase, nitrogenase and haloperoxidase [5] but also acts as a prooxidant that increases oxidative stress [6]. Since both the mobility and toxicity of vanadium increase with its oxidation state, the reduction of vanadate to lower oxidation states is a critical step in the biogeochemical vanadium cycle and in the bioremediation of vanadium contamination [3, 4]. However, reduction by chemical agents is often slow and not cost-effective [7, 8], underscoring the importance of microbially mediated reduction processes.

The microbial reduction of vanadate can be coupled to energy-conserving respiratory electron transport or alternatively represent a detoxification process. The vast majority of vanadate reducers identified to date are respiratory microorganisms with the capacity to utilize metals or nitrate as electron acceptors and belong to the phylum Proteobacteria [9]. In most environments, vanadate concentrations are rather low, limiting the potential for these microorganisms to derive a significant energetic benefit from the respiratory reduction of vanadate [10], and the anaerobic respiration process also competes with fermentation for available organic substrates. Thus, fermentative microorganisms that are tolerant to vanadate and capable of detoxifying it, if present, may play a significant role in the biogeochemical vanadium cycle. However, research on fermentative reduction has lagged behind that on vanadate respiration. While respiratory vanadate reduction is limited to Gram-negative bacteria, the dominance of Gram-positive fermenters has been reported in vanadate-amended sludge cultures [11], but the fermentative bacterium *Lactococcus raffinolactis* remains the only Gram-positive bacterium shown to reduce vanadate in pure culture [12]. Consequently, further investigations within a broader phylogenetic and physiological context are warranted for a better understanding of microbial vanadate reduction in both natural and engineered environments.

In this study, we report vanadate reduction by the strain VROV1 of *Tepidibacter mesophilus*, which was isolated from deep-sea sediments on the northern Central Indian Ridge. This strain is the second Gram-positive vanadate reducer identified, and the first within the class *Clostridia*. Although, as an obligate fermenter, the strain VROV1 is unlikely to couple vanadate reduction to energy conservation, we demonstrate that vanadate reduction is a direct result of biological activity rather than a result of secondary reactions with metabolic waste products. The rate of vanadate removal is comparable to that reported for respiratory vanadate reducers. Several species within the *Tepidibacter* genus have been previously proposed as bioremediation agents for metal contamination [13, 14], and the discovery of vanadate-reducing capabilities within this genus expands the role of these fermentative microorganisms in the cycling of metals or metalloids.

## Methods

### Isolation and cultivation

A vanadate-reducing strain was isolated from deep-sea surface sediments of the Onnuri Vent Field near the Central Indian Ridge, located in international waters at 11°24.9′S, 66°25.4′E [15]. The sediments were collected with a TV grab at a depth of 2,022 m and prepared into slurries with deoxygenated artificial seawater [16]. An enrichment culture was performed in the following carbonate-buffered medium: NaCl (21 g/L), NaHCO_3_ (9 g/L), NH_4_Cl (1 g/L), KH_2_PO_4_ (0.5 g/L), MgCl_2_ (1.4 g/L), CaCl_2_ (0.11 g/L), NaVO_3_ (0.61 g/L), sodium citrate (0.3 g/L), yeast extract (1 g/L), sodium lactate solution (60% w/w) (0.7 mL/L), and trace element solution (SL-10) [17]. The medium was prepared anaerobically under 80% N_2_/20% CO_2_ with a pH of 7.4. Following several transfers at room temperature, the final enrichment culture was serially diluted 10-fold to 10^−10^ in fresh medium. The highest dilution that turned a turquoise blue color corresponding to vanadyl ions was used as an initial inoculum, and this dilution-to-extinction cultivation was repeated 10 times until only one cell morphology was observed under a microscope. One isolate designated VROV1 was selected, and its purity and phylogenetic identity were further examined via 16S rRNA gene sequencing. DNA was extracted and purified with the Wizard Genomic DNA Purification Kit (Promega). PCR amplification was subsequently carried out on a MiniAmp Thermal Cycler (Applied Biosystems) using the bacterial 16S rDNA PCR Kit (Takara), and the amplicon was sequenced at the National Instrumentation Center for Environmental Management (NICEM), Seoul National University. The obtained sequence (NCBI accession number PQ219474) was subjected to a similarity search through NCBI BLAST, and a phylogenetic tree was constructed using the neighbor-joining method in MEGA X software [18].

After establishing the pure culture, the strain was grown in fully defined medium unless otherwise specified, in which yeast extract (1 g/L) was substituted with 10 mL of vitamin solution from DSMZ medium 141, 10 mL of MEM nonessential amino acid solution (Thermo Fisher Scientific), and 1 mL of selenium stock solution (0.4 mg of Na_2_SeO_4_ per 200 mL of 0.01 N NaOH) per liter. Changes in cell density and morphology were monitored under a fluorescence microscope (Echo Revolve) after the cells were stained with SYTOX-Green (Invitrogen). Potential electron donors or fermentation substrates screened for their ability to support growth and reduce vanadate included acetate, fructose, glucose, lactate, pyruvate, alanine, isoleucine, leucine, serine, tryptophan, valine (all 10 mM), MEM amino acid solution (10 mL/L), yeast extract (1 g/L) and beef extract (1 g/L). In addition to vanadate, nitrate and sulfate were tested as potential electron acceptors.

### Characterization of vanadium metabolism

The vanadate concentration in aqueous solution was measured using the diphenylcarbazide (DPC) assay [19, 20]. DPC reacts with pentavalent vanadate but not with tetravalent or trivalent vanadium, allowing for the quantification of microbial vanadate reduction. The total dissolved vanadium content was determined via ICP‒AES (Optima 8300, PerkinElmer) at the National Center for Interuniversity Research Facilities (NCIRF), Seoul National University. The amount of vanadium accumulated in the solid phase was also quantified by ICP‒AES after the precipitates were collected via centrifugation and dissolved in 1 M HCl. The oxidation state of the precipitated vanadium was determined by XPS analysis using NaVO_3_, VOSO_4_ and VCl_3_ as references for pentavalent, tetravalent and trivalent vanadium, respectively, at the Central Laboratory of Pukyung National University. To determine whether vanadate reduction was directly mediated by the biological processes of the isolated strain or occurred via chemical reactions with its metabolic products, an early stationary phase culture in vanadium-free medium was sterilized using a 0.2 μm pore syringe filter. Then, 3 mL of the filtrate was transferred to 30 mL of fresh medium containing vanadate. The influence of volatile metabolites was also examined by replacing 40% of the headspace of the fresh vanadate-containing medium with that of the early stationary phase culture, either with or without vanadate. In both experiments, vanadate reduction was monitored by the DPC assay.

Samples for identification and quantification of organic compounds were collected by filtering 1 mL of culture through a 0.2 μm syringe filter and stored at -80 °C until use. Simple organic acids, alcohols, and carbohydrates were analyzed via a Dionex Ultimate 3000 HPLC system (Thermo Fisher Scientific) equipped with a UV detector at 210 nm and a refractive index detector. Separation was achieved on an Aminex 87H column (Bio-Rad) with 0.01 N H_2_SO_4_ as the isocratic mobile phase at 0.5 mL/min. Amino acids were analyzed after derivatization with o-phthalaldehyde (OPA) and fluorenylmethyl chloroformate (FMOC) via a Dionex Ultimate 3000 HPLC system (Thermo Fisher Scientific) with a fluorescence detector. The derivatized amino acids were separated on an Inno C18 column (Youngjin Biochrom) by gradient elution using phosphate buffer (pH 7) and a mixture of acetonitrile and methanol as the mobile phases. Both HPLC analyses were carried out at NICEM. The headspace gas sample was analyzed via a gas chromatograph (Agilent 7980B) equipped with a thermal conductivity detector and an HP-Molsieve column (Agilent). The oven temperature was set to 50 °C, and Ar was used as the carrier gas.

## Results

**Fig 1:**
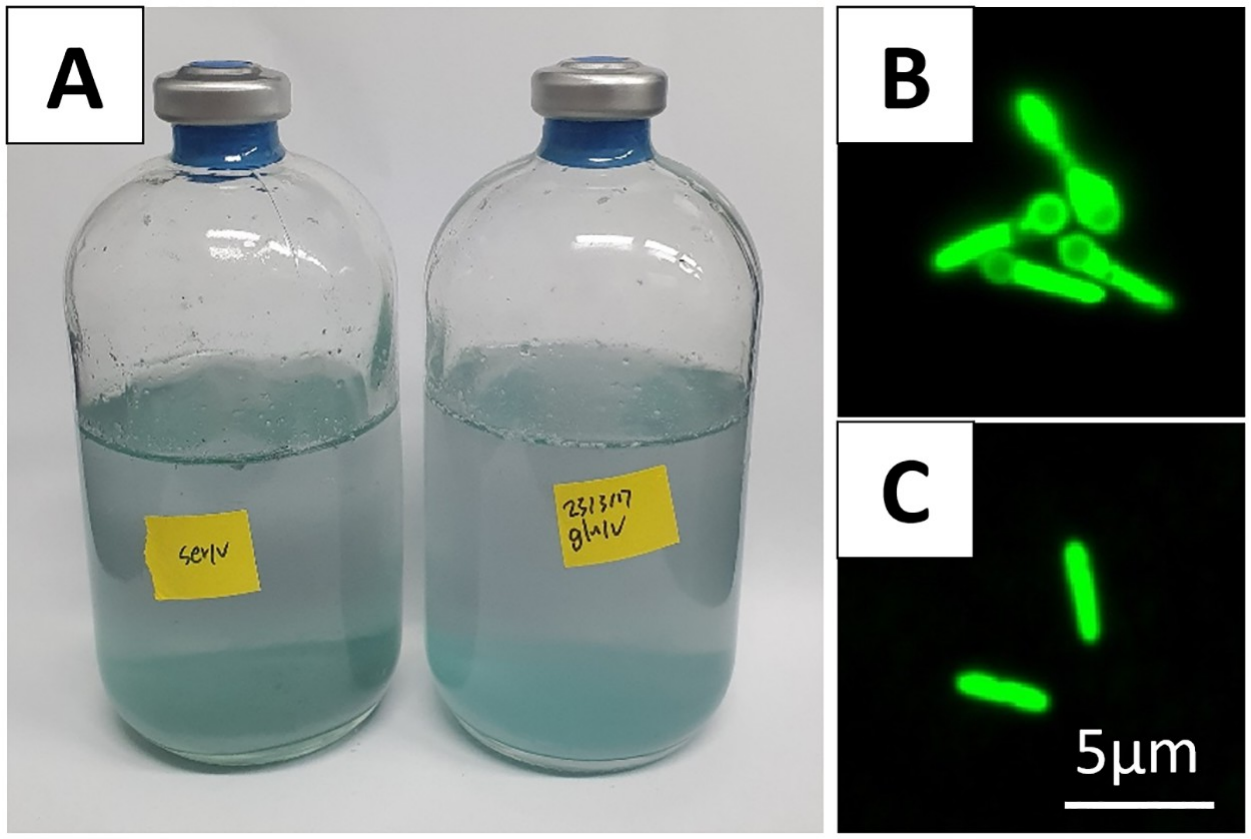
Photographs of strain VROV1 isolated from marine sediments collected on the northern Central Indian Ridge. (A) A typical turquoise–blue color developed in vanadate-reducing VROV1 cultures. (B) Epifluorescence photomicrograph of bacterial cells entering the stationary phase and initiating endospore formation. (C) Epifluorescence photomicrograph showing vegetative cells in the exponential growth phase.

**Fig 2:**
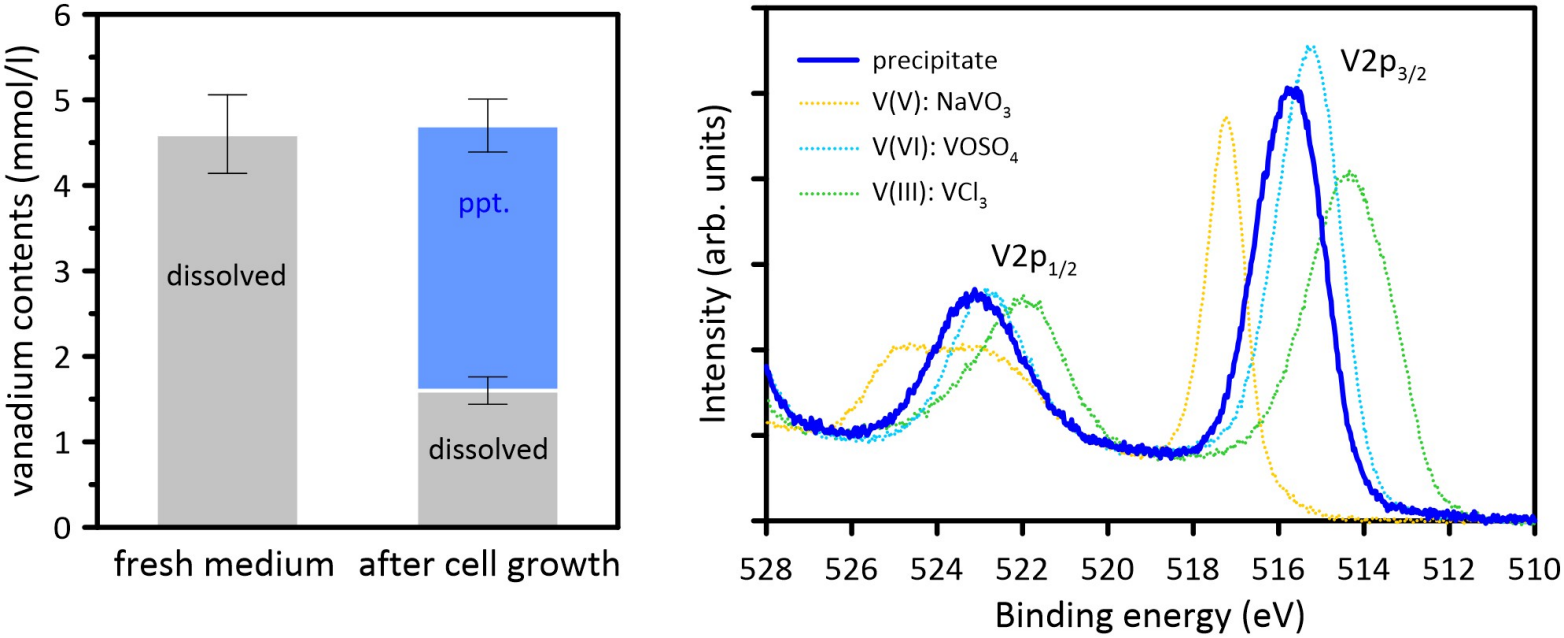
Reductive precipitation of vanadium by strain VROV1. The removal of vanadium due to precipitation accounts quantitatively for the decrease in dissolved vanadium (left). Comparison of the V2p XPS spectra between culture precipitate and reference vanadium compounds confirmed the biological reduction of vanadate to vanadyl (right). The relative error in vanadium determination by ICP‒AES was 10%. Abbreviations: ppt., precipitate; arb., arbitrary.

The newly isolated strain VROV1 was characterized as an anaerobic, Gram-positive bacterium capable of reducing vanadate at millimolar levels. It forms endospores, as depicted in Fig 1. Incubation experiments conducted at temperatures ranging from 4 to 84 °C demonstrated that the strain grows optimally at room temperature (25 °C). VROV1 fermented a wide variety of substrates, including sugars, simple organic acids, amino acids, and complex organic mixtures such as yeast and beef extracts (Table 1). In the presence of vanadate at concentrations up to 5 mM in the medium, fermentable substrates supported growth, and as microbial growth progressed, the medium turned light greenish-blue in color, indicating the reduction of vanadate to vanadyl ion (Fig 1A). No vanadate reduction was observed in the control experiments (S1 Fig; S1 Table). Precipitates of a similar bluish color were also formed, and these precipitates account for the reduction in dissolved vanadium concentration in the culture medium (Fig 2A; S1 Table). The oxidation state of vanadium in the solid phase was subsequently examined by analyzing the binding energy of the V2p_3/2_ peak in the XPS spectrum. The literature values for the V2p_3/2_ XPS lines of VO_2_ and V_2_O_5_ are 515.84 eV and 517.84 eV, respectively [21], and the V2p_3/2_ signal of VOSO_4_, used as a reference for V(IV) in this study, was found at 515.18 eV. The V2p_3/2_ binding energy for the culture precipitates at 515.57 eV thus indicated that the majority of vanadium in the solid phase was composed of V(IV) (Fig 2B; S1 Table).

**Table 1:** Growth and vanadate reduction in basal medium with specific supplements.

Supplement		Growth	Vanadate reduction	
			Without cells	With cells
none		na	-	na
Reducing agents	sulfide		+	
	titanium (III)		+	
	thioglycolate		+	
	ascorbate		+	
Metabolic products	molecular hydrogen		-	
	recycled culture medium		-	
	recycled headspace gas		-	
Substrates*	acetate	-	-	-
	fructose	+	-	+
	glucose	+	-	+
	lactate	-	-	-
	pyruvate	+	-	+
	alanine	-	-	-
	isoleucine	+	-	+
	leucine	+	-	+
	serine	+	-	+
	tryptophan	-	-	-
	valine	+	-	+
Complex nutrient mixtures	MEM amino-acid mixture	+	-	+
	yeast extract	+	-	+
	beef extract	+	-	+

* A trace amount of either MEM mixture or yeast extract was also added as a source of micronutrients.

na, not applicable; +, growth or vanadium reduction occurred; -, no growth occurred or vanadium reduction occurred.

Potential abiotic pathways exist for the reductive precipitation of vanadate because reducing agents commonly used in anaerobic growth media, such as sulfide or ascorbate, are capable of reducing vanadate (Table 1). However, these reducing agents were not present in the culture media used in this study, and no vanadate reduction occurred in fresh media without inoculation (Table 1), verifying that the chemical components in the media did not serve as abiotic reductants for vanadate reduction. To distinguish between direct microbial reduction and indirect reduction by metabolic products, dissolved and volatile components of VROV1 cultures grown in vanadate-free medium were filter-sterilized and added to a medium containing 5 mM vanadate, and no change in the dissolved vanadate concentration was observed. Replacing the headspace of the fresh medium with H_2_, a possible fermentation product, also did not induce vanadate reduction. These results confirmed that vanadate reduction was directly mediated by the biological processes of the newly isolated strain VROV1 (Table 1).

**Fig 3:**
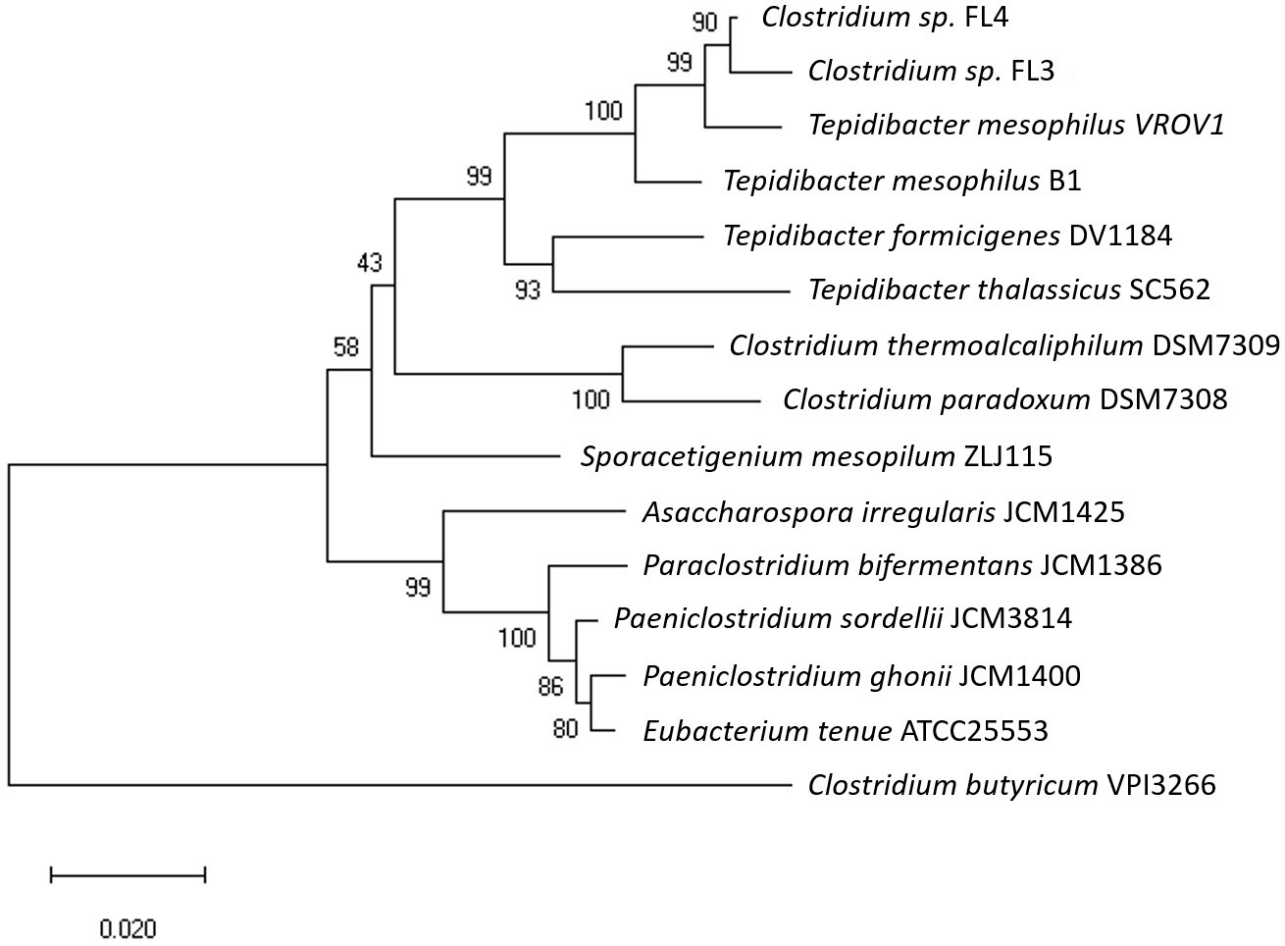
Neighbor-joining phylogenetic tree of 16S rRNA gene sequences showing the relationship of *T*. *mesophilus* VROV1 to representative species within the order *Clostridiales*. Node support was evaluated with 1,000 bootstrap replicates.

Phylogenetic analysis of the 16S rRNA gene revealed that vanadate-reducing VROV1 belongs to the genus *Tepidibacter*, which is located within cluster XI of *Clostridia* (Fig 3) [22]. The closest matches were *Clostridium* sp. FL3 and FL4, which were isolated from the Japan Trench [23], and the next closely related strain was *Tepidibacter mesophilus* B1^T^, which was isolated from oil-contaminated soil in China [24]. The 16S rRNA gene sequence similarity between VROV1 and *T*. *mesophilus* B1^T^ was 98.68%. These strains share some physiological characteristics with VROV1, including mesophilic growth, spore formation, and fermentation of certain sugars, but none have been examined for their capacity to reduce vanadate or to grow by fermenting amino acids. Furthermore, while its close relatives were grown in complex media containing chemically undefined components such as yeast extract, the VROV1 cultures were established and maintained in a fully defined medium, allowing for a more systematic assessment of its metabolic processes.

**Fig 4:**
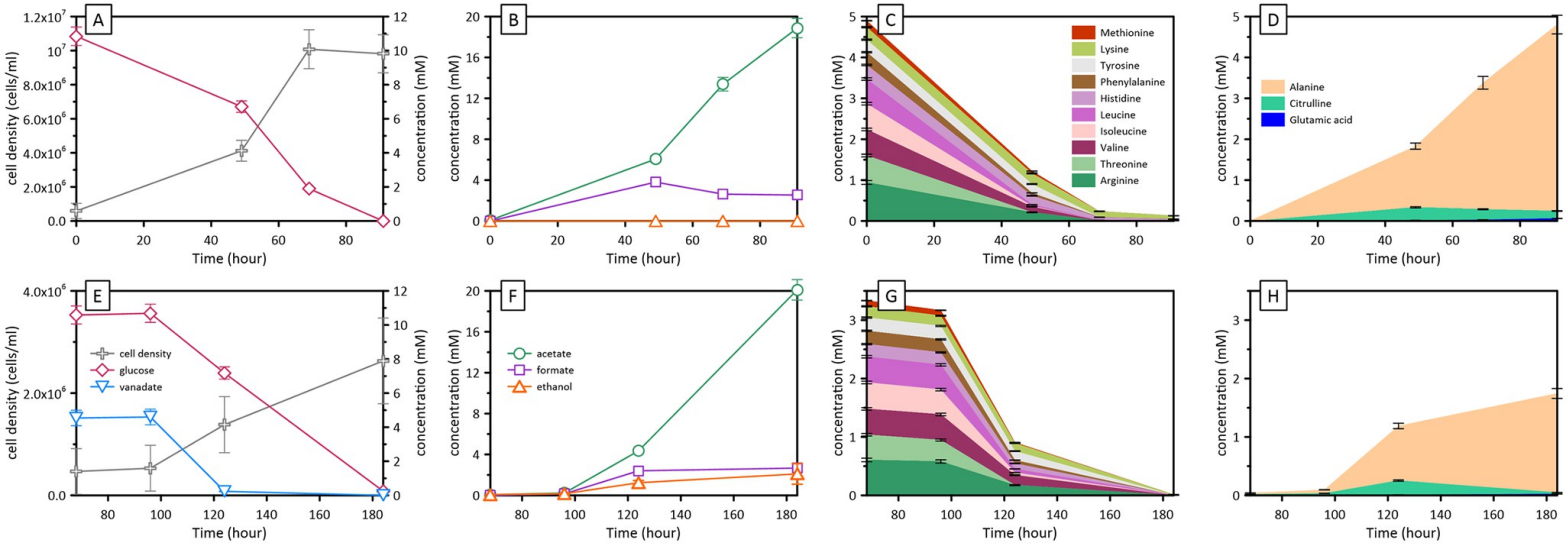
Growth and metabolic characteristics of strain VROV1 in vanadate-free (upper panels) and vanadate-containing (lower panels) media. Growth and consumption of glucose and vanadate (A, E). Production of primary metabolites excluding amino acids (B, F). Consumption of amino acids in a medium supplemented with a MEM amino acid mixture (C, G). Production of amino acids not included in the MEM amino acid mixture (D, H). Note the prolonged lag phase in the presence of vanadate. The error bars for the cell density reflect the standard deviation of the cell counts, whereas the concentrations determined via colorimetry and chromatography are subject to analytical errors of ±10% and ±5%, respectively.

Although a wide range of organic compounds can be utilized as carbon and energy sources by VROV1, the effect of vanadate on the metabolic processes of VROV1 was examined using a medium containing glucose as the primary substrate supplemented with MEM amino acid solution (Fig 4; S1 Table). In the vanadate-free medium, the concentrations of glucose and MEM amino acids decreased with increasing cell density up to 10^7^ cells/mL (Fig 4A). Acetate was the most abundant carboxylic acid produced during fermentation, followed by formate (Fig 4B). Alanine constituted the majority of the amino acids produced, and its concentration was comparable to the sum of the MEM amino acids consumed during fermentation (Fig 4C and 4D). No H_2_ production was detected via headspace gas analysis. In the culture supplemented with 5 mM vanadate, the lag phase was extended, and the final cell density decreased by a factor of 3 compared with that of the vanadate-free culture (Fig 4E). Vanadate reduction involved changes in other catabolic processes. In addition to acetate and formate, ethanol accumulated in the medium during fermentation (Fig 4F). Alanine production also declined significantly, accounting for only approximately half of the MEM amino acids consumed (Fig 4G and 4H). The reduction in alanine production was replicated in the presence of 1 mM vanadate (S2 Fig; S1 Table). Not shown in Fig 4, a trace amount of H_2_ was detected in the headspace, corresponding to less than 0.2 mM if all the H_2_ in the headspace was dissolved in the medium.

## Discussion

### Vanadium metabolism by VRVO1

**Fig 5:**
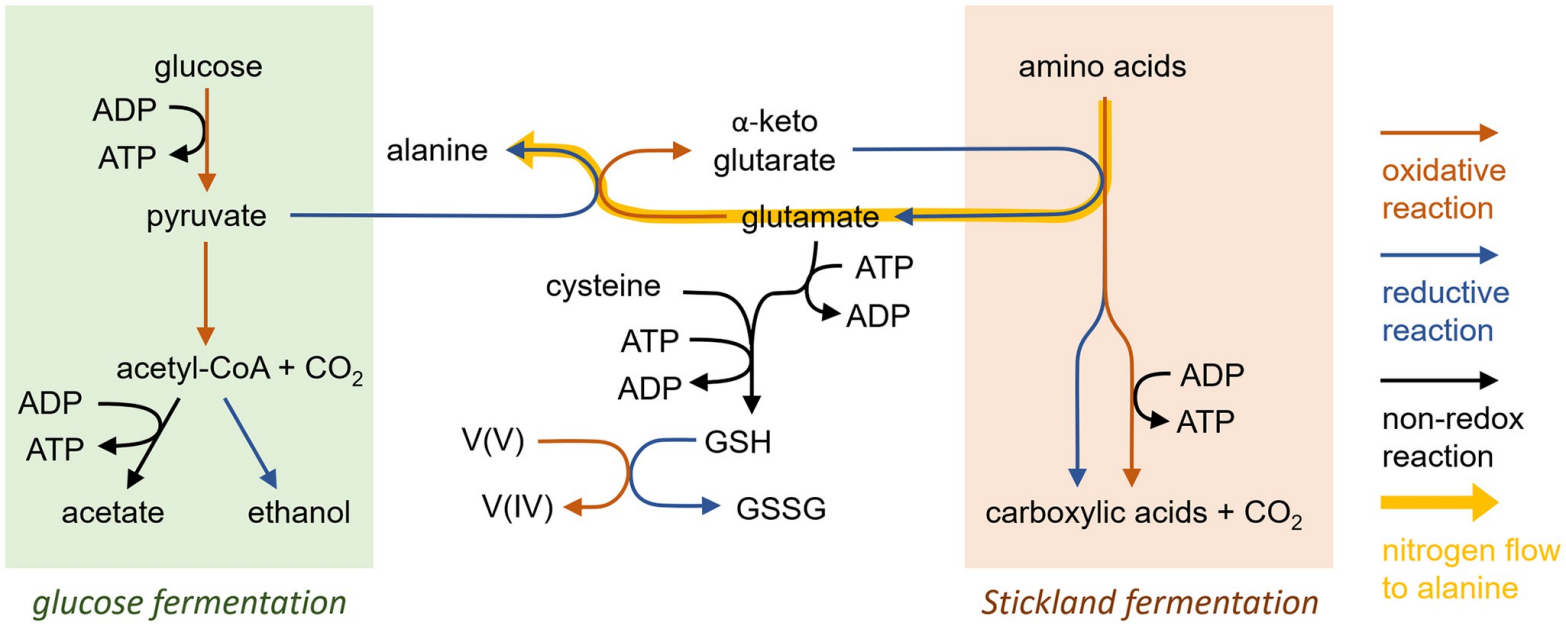
Proposed metabolic pathways related to vanadate reduction and alanine production by *T*. *mesophilus* VROV1. The transamination of pyruvate to alanine bridges glycolysis and Stickland fermentation, maintaining the redox and nitrogen balances of overall fermentation reactions [31]. A metabolic shunt toward vanadate reduction can disrupt this coupling.

Our results confirmed that the strain VROV1, which was isolated from deep-sea sediments, is not only a fermenter but is also capable of reducing vanadate through its metabolic processes. Among named species, VROV1 is most closely related to the Gram-positive *T*. *mesophilus* (Fig 3), while the majority of vanadate-reducing prokaryotes reported in the literature are Gram-negative metal reducers, largely represented by the genera *Geobacter* and *Shewanella* (Table 2) [10, 25]. Recently, an increasing number of Gram-negative denitrifying bacteria have also been demonstrated to possess the capacity to reduce vanadate [9]. A few other bacterial strains, including those belonging to the genera *Acidothiobacillus*, *Halomonas* and *Polaromonas*, are able to reduce vanadate [26–28]. To date, however, the reduction of vanadate by a pure culture of Gram-positive bacteria has been demonstrated only in the fermentative bacterium *Lactococcus raffinolactis* [12], which is taxonomically distinct from *T*. *mesophilus* at the class level. Gram-negative metal-reducing bacteria, such as *Shewanella oneidensis*, use membrane-localized menaquinones and cytochromes to facilitate electron transfer to vanadate, precipitating reduced vanadium products at the cell surface [10], and the roles of dissimilatory nitrate and nitrite reductases in vanadate reduction have been verified in denitrifying bacteria [9, 29]. The genes for dissimilatory iron or nitrate reduction have not been identified in the genome of *T*. *mesophilus* [30], but intracellular NADH, glutathione, and riboflavin have been proposed as potential electron donors for vanadate reduction [9, 11]. Further investigation is needed to ascertain the pathway and physiological consequences of vanadate reduction in VROV1; however, the shift in metabolite production in response to the presence or absence of vanadate indicates that vanadate may disrupt the reactions linking glycolysis and amino acid fermentation (Figs 4 and 5).

**Table 2:** Comparison of vanadate reduction by different bacterial strains.

Microorganism	Gram staining	Class	Vanadate concentration	Organic substrate	Reduction rate (mM/day)	^#^Specific rate (pmol/cell/day)	Reference
*Tepidibacter mesophilus* VROV1	positive	*Clostridia*	5 mM	glucose	3.7	2.8	this study
*Lactococcus raffinolactis*	positive	*Bacilli*	1 mM	citrate	0.09	0.16	[12]
*Shewanella oneidensis*	negative	*Gammaproteobacteria*	5 mM	lactate	4.8	1.6	[10]
*Halomonas* strain Mono	negative	*Gammaproteobacteria*	4 mM	acetate	0.82	-	[27]
*Geobacter metallireducens*	negative	*Deltaproteobacteria*	1 mM	acetate	0.42	0.07	[25]
*Acidovorax* sp. strain BoFeN1	negative	*Betaproteobacteria*	0.8 mM	acetate	0.001	0.02	[9]
*Pseudogulbenkiania* sp. strain 2002	negative	*Betaproteobacteria*	0.8 mM	acetate	0.001	0.04	[9]

^#^Calculated assuming OD600 of 1 is equals 8 * 10^8^ cells/mL, where only optical density was measured

In the vanadate-free culture supplemented with glucose and MEM amino acids, VROV1 produced alanine as one of the primary metabolic products (Fig 4). The amino nitrogen of the produced alanine can account for the majority of the ammonia released by the oxidative deamination of MEM amino acids (Fig 4). As has been reported for related *Clostridium* strains, alanine production occurred likely due to the transamination of pyruvate, the product of glycolysis, with ammonia derived from MEM amino acids (Fig 5) [31–33]. This transamination reaction is coupled with the deamination of glutamate to α-ketoglutarate, where no reducing equivalents are produced or consumed. Conversely, the fermentative oxidation of pyruvate to acetyl-CoA generates reducing equivalents, which must be balanced to maintain the cellular redox state and metabolic flow (Fig 5). By bridging glycolysis and amino acid fermentation, *T*. *mesophilus* VROV1 might increase energy generation and nitrogen handling, similar to other *Clostridium* strains [31]. In the presence of vanadate, however, VROV1 presented lower alanine yield than the amount of MEM amino acids consumed, which was compensated for by increased ethanol production (Fig 4). This suggests that a larger fraction of pyruvate was oxidized to acetyl-CoA rather than transaminated by glutamate, and the resulting accumulation of reducing equivalents was partially mitigated by the reduction of acetyl-CoA to ethanol. Notably, glutamate serves not only as an amino group donor for the transamination reaction but also as a precursor for the synthesis of glutathione, which is involved in the reduction and detoxification of heavy metals [34, 35]. In fact, anaerobic sludge dominated by the Gram-positive classes *Bacilli* and *Clostridia* produced more intracellular glutathione during the reduction of vanadate [11]. Therefore, a high demand for glutamate for glutathione synthesis triggered by vanadate likely shifted the metabolic flux from the transamination shunt to mixed acid fermentation (Fig 5). The marked reduction in microbial growth during vanadate reduction may be explained by the fact that glutathione synthesis consumes ATP (Fig 5). Although the primary function of vanadate reduction appears to be the alleviation of oxidative stress, rather than energy generation, the fermentative strain VROV1 can tolerate vanadate concentrations as high as 5 mM and eliminate vanadate as effectively as metal- or nitrate-reducing microorganisms (Table 2). For example, when cultured in the presence of 5 mM vanadate, the metal-reducing agent *S*. *oneidensis* reduced vanadate at a rate of approximately 1.6 pmol/cell/day [10], whereas at a similar vanadate concentration, the rate of vanadate reduction by *T*. *mesophilus* VROV1 exceeded 2 pmol/cell/day (Table 2). During fermentative growth at a vanadate concentration of 1 mM, *L*. *raffinolactis*, the only Gram-positive vanadate reducer cultivated prior to this study, showed a specific rate approximately 10 times slower than that of VROV1 (Table 2) [12].

### Biogeochemical vanadium cycle and bioremediation

The newly isolated strain VROV1 expands the list of highly effective microbial agents for vanadate reduction to include Gram-positive fermenters, providing new insights into biogeochemical vanadium transformation in both engineered and natural systems.

Metals and metalloids, including vanadium, play pivotal roles in numerous enzymatic reactions but become toxic at higher concentrations, posing significant environmental risks [36]. In response to elevated levels of these elements, microorganisms may employ either or both of the two primary mechanisms: transforming the pollutant into a less toxic form or developing resistance [37]. This aligns with the observed response of VROV1 to high concentrations of vanadate. Given that the mobility and toxicity of vanadium are highly dependent on its oxidation state, with pentavalent vanadium posing a greater environmental risk, microbial reduction and precipitation of vanadium have been considered promising approaches for the bioremediation of vanadate-polluted environments [29]. While the addition of organic electron donors is a common technique to stimulate the microbial reduction of metal or metalloid contaminants, competition for organic substrates between respiratory and fermentative microorganisms can impair bioremediation efficacy, particularly when fermentation does not involve the reduction of contaminants [38, 39]. Nevertheless, the rapid vanadate reduction by the pure culture of *T*. *mesophilus* VROV1 demonstrated that in vanadium-contaminated environments, fermentative microorganisms may not impede bioremediation but rather play a substantial role in the overall bioremediation process. This finding is consistent with the reported predominance of Gram-positive fermenters in vanadate-amended anaerobic sludge [11].

On a global scale, vanadium ranks as the second most abundant trace element in modern oxygenated seawater, with riverine input representing a primary source of vanadium to the ocean [40]. Adsorption by Fe‒Mn oxides in marine sediments and hydrothermal plumes is generally considered the major mechanism responsible for the removal of vanadate from oxygenated seawater, whereas a reductive sink constitutes a minor pathway [41, 42]. However, we demonstrated that the fermentative *T*. *mesophilus* VROV1, which was isolated from oceanic sediments, can effectively reduce and eliminate vanadium from the dissolved phase. The *T*. *mesophilus* strain B1^T^ was first isolated from oil-polluted soil in China [24]; moreover, the recovery of *T*. *mesophilus* VROV1 from the Central Indian Ridge highlights the adaptability of *T*. *mesophilus* and its potential to disperse across diverse environments. If reduction occurs due to reactions with metabolic waste products, the loss of reductive metabolites through diffusion or adsorption can compromise the environmental significance of fermentative vanadate reduction. However, this is not the case for VROV1 (Table 1). Moreover, the stimulation of fermenters does not depend on the availability of inorganic electron acceptors such as nitrate or ferric iron. Although speculative at this stage, we suggest that fermentative vanadate reduction may be widespread in marine sediments and account for a nonnegligible reductive sink in the biogeochemical cycle of vanadium.

## Conclusions

This study demonstrates that *T*. *mesophilus* VROV1, which was isolated from deep-sea sediments, is capable of reducing vanadate while fermenting a variety of organic substrates, including amino acids. Vanadate reduction and subsequent precipitation can be considered a direct consequence of biological mediation, as opposed to indirect chemical processes involving metabolic byproducts. As the second Gram-positive bacterium identified to reduce vanadate, VROV1 broadens the diversity of vanadate reducers to include members of the class *Clostridia*. This reduction appears to serve primarily as a detoxification process rather than contributing to energy production. Although the precise mechanism remains to be elucidated, vanadate reduction significantly alters carbon and nitrogen metabolism, shifting the overall metabolic balance from transamination towards mixed acid fermentation. With its superior ability to rapidly reduce high concentrations of vanadate, comparable to the metal-reducing *Shewanella oneidensis*, VROV1 shows promise for addressing vanadium contamination through bioremediation. Furthermore, the presence of closely related strains in diverse habitats suggests that fermentative vanadate reduction may be widespread in marine sediments, potentially representing an underexplored reductive sink in the biogeochemical cycling of vanadium. Future research should focus on assessing the ecological significance of this process in natural environments and its practical applications in bioremediation.

## Supporting information

S1 Fig Dissolved vanadate concentration in culture and control experiments over time. No change in vanadate concentration was observed in uninoculated medium. The error bars represent analytical reproducibility (±10%).
(TIF)

S2 Fig Consumption (left) and production (right) of amino acids by strain VROV1 in medium containing 1 mM vanadate. The error bars represent analytical reproducibility (±5%).
(TIF)

S1 Table All data underlying the figures in the main text and supporting information. (XLSX)
