# Supplementary figures and images for: Vanadate reduction by gram-positive fermentative bacteria isolated from deep-sea sediments on the northern Central Indian Ridge
Source: PLoS One. 2025 Jan 22;20(1):e0317320. doi: 10.1371/journal.pone.0317320 (PMC11753629; doi:10.1371/journal.pone.0317320)

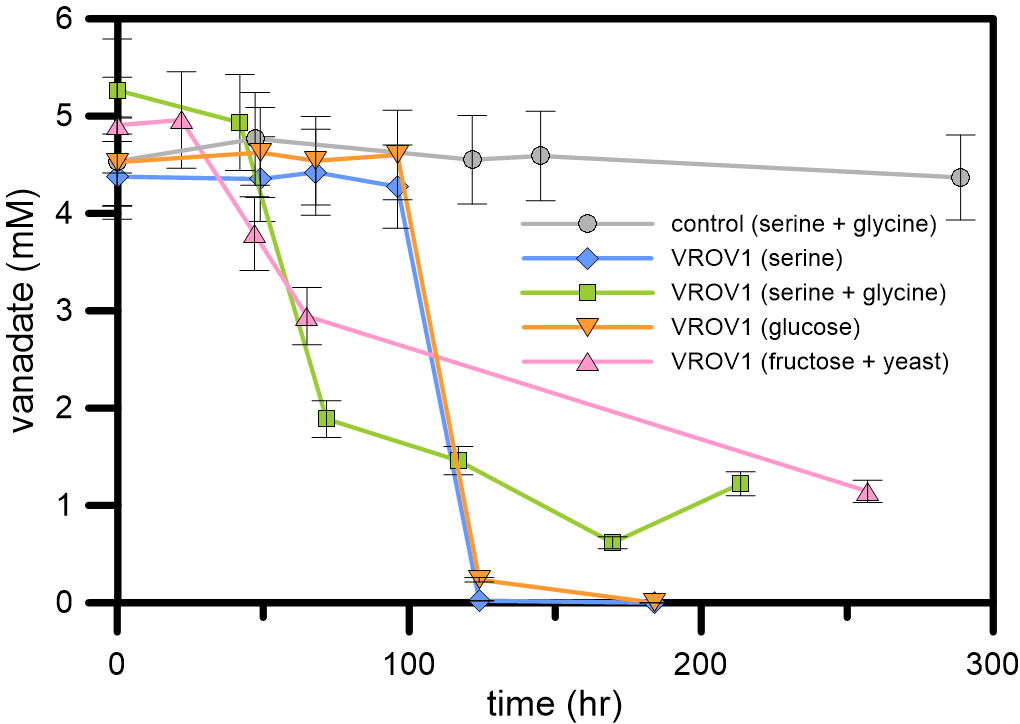

Supplement: S1 Fig — No change in vanadate concentration was observed in uninoculated medium. The error bars represent analytical reproducibility (±10%). (TIF) [file pone.0317320.s001.tif]

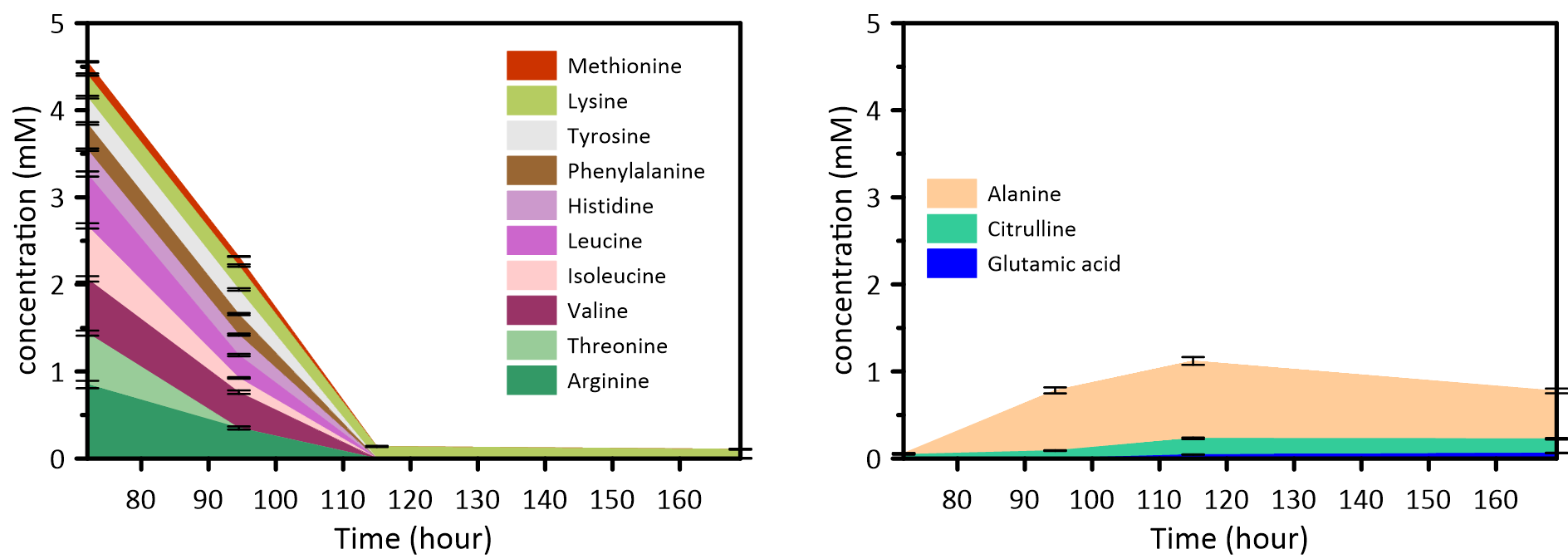

Supplement: S2 Fig — The error bars represent analytical reproducibility (±5%). (TIF) [file pone.0317320.s002.tif]
